# Targeting Azole-Resistant *Candida albicans*: Tetrapeptide Tuftsin-Modified Liposomal Vaccine Induces Superior Immune Protection

**DOI:** 10.3390/vaccines13060630

**Published:** 2025-06-11

**Authors:** Masood A. Khan, Arif Khan, Abdullah M. Alnuqaydan, Aqel Albutti, Basmah F. Alharbi, Mohammad Owais

**Affiliations:** 1 Department of Basic Health Sciences, College of Applied Medical Sciences, Qassim University, Buraydah 51412, Saudi Arabia; 4140@qu.edu.sa (A.K.); ami.alnuqaydan@qu.edu.sa (A.M.A.); as.albutti@qu.edu.sa (A.A.); b.alwahbi@qu.edu.sa (B.F.A.); 2 Interdisciplinary Biotechnology Unit, Faculty of Life Sciences, Aligarh Muslim University, Aligarh 202002, India

**Keywords:** vaccination, antibodies, *C. albicans*, immunity, toxicity, cytokine

## Abstract

Background/objectives: *Candida albicans* is a major fungal pathogen that poses a serious threat to immunocompromised individuals. The increasing prevalence of fluconazole-resistant strains presents a critical clinical challenge, emphasizing the urgent need for novel therapeutic strategies. This study aimed to evaluate the prophylactic potential of a new liposomal vaccine formulation, Tuft-lip-WCAgs, comprising Tuftsin and *C. albicans* whole cell antigens, in providing immune protection against systemic candidiasis. Methods: The vaccine formulation was tested in a murine model of systemic *C. albicans* infection. The efficacy of the Tuft-lip-WCAg vaccine was evaluated through a survival analysis, fungal burden assessments, and immunological profiling. Immune responses were assessed by measuring serum antibody titers and isotypes, T cell proliferation, and cytokine secretion (IFN-γ and IL-4) from splenocytes. Results: FLZ treatment showed weak antifungal activity, high MIC values, and limited biofilm disruption and failed to ensure long-term survival, resulting in 100% mortality by day 40. In contrast, Tuft-lip-WCAg vaccination was well tolerated and conferred complete protection, with no detectable fungal burden by day 40. Vaccinated mice exhibited significantly elevated total antibody titers (166,667 ± 14,434), increased IgG2a levels, and enhanced T cell proliferation (stimulation index: 3.9 ± 0.84). Splenocytes from immunized mice secreted markedly higher levels of IFN-γ (634 ± 128 pg/mL) and IL-4 (582 ± 82 pg/mL), indicating a balanced Th1/Th2 immune response. Tuft-lip-WCAg vaccination also achieved 100% survival and the lowest kidney fungal burden (556 ± 197 CFUs/g). Conclusions: Tuft-lip-WCAg formulation is a safe, immunogenic, and highly effective vaccine candidate that offers complete protection against drug-resistant *C. albicans* in a murine model. These findings support its promise as a novel immunoprophylactic strategy, particularly for immunocompromised populations at high risk of invasive candidiasis.

## 1. Introduction

*Candida* species rank among the leading fungal pathogens, posing a significant threat to those with impaired immune defenses [1,2]. Conditions such as HIV/AIDS, organ transplantation, or prolonged immunosuppressive therapy can impair the host’s ability to control fungal infections [3]. Among antifungal agents, polyenes and azoles are frequently used for treating fungal diseases. Although amphotericin B remains one of the most potent antifungal agents, its clinical utility is limited due to significant toxicity and high cost [4]. Interestingly, liposomal amphotericin B has been shown to reduce its toxicity considerably [5]. The overuse of azole antifungals has facilitated the development of resistant *Candida* strains and cross-resistance to multiple antifungal therapies [6,7]. Effective clearance of *Candida* in immunocompromised patients relies on coordinated responses by neutrophils, macrophages, and T cells [8,9,10].

Immunotherapeutic strategies have shown promise in combating fungal infections at the population level [11,12]. Th1 and Th2 responses have been shown to influence both the pathogenesis and immune regulation of vulvovaginal candidiasis (VVC) [13]. To date, no vaccine has been approved for active immunization against *Candida* infections in humans. However, earlier studies have identified immunogenic *Candida* components such as cell wall polysaccharides, mannans/mannoproteins, and intracellular proteins that can elicit partial protection [9,14,15,16]. Immunization with cell wall mannoproteins has demonstrated efficacy against systemic candidiasis, although some immunosuppressive effects have been reported [17]. In contrast, cytosolic fractions, lacking cell wall components, may have fewer adverse immunomodulatory effects. Studies have characterized cytoplasmic antigens for their immunogenicity, including a major antigen released during infection by *C. albicans* [18]. Despite progress, only two vaccine formulations have shown limited success in conferring protection against candidiasis [19,20]. For instance, a vaccine based on a recombinant adhesin/invasin protein reduced the severity of VVC in women [21]. Vaccine candidates such as NDV-3A, Sap2-based formulations, and oral immunization with recombinant enolase 1 have all been shown to elicit strong immune responses against *Candida albicans* [22,23,24].

Resistance to azoles in *C. albicans* poses a serious clinical challenge, particularly in immunocompromised patients who rely on antifungal therapy for managing systemic or recurrent infections [25]. By targeting lanosterol 14α-demethylase (ERG11), azoles interfere with ergosterol synthesis and compromise fungal membrane integrity [26]. However, *C. albicans* has evolved multiple resistance mechanisms, including *ERG11* mutations, upregulation of efflux pumps (*CDR1*, *CDR2*, and *MDR1*), and alterations in the ergosterol biosynthetic pathway [26]. Biofilm formation further complicates treatment, as sessile *C. albicans* cells exhibit enhanced tolerance to azoles as compared to planktonic cells [27].

This growing resistance trend emphasizes the urgent need for novel antifungal approaches, including new drug targets, combination therapies, and vaccines [28]. Liposomes have gained attention as promising antigen delivery systems in vaccine development against fungal, bacterial, viral, and protozoan infections [14,29,30]. Their efficient antigen delivery to APCs, including macrophages and dendritic cells, makes them ideal immune response enhancers [31]. Tuftsin, a tetrapeptide derived from the Fc region of IgG (residues 289–292), has been shown to enhance liposomal efficacy in both bacterial and fungal infections [32]. It enhances innate immune responses by promoting the phagocytic activity of macrophages and neutrophils and stimulates antigen presentation by dendritic cells [33]. Tuftsin also facilitates microbial clearance by boosting oxidative burst and increasing cytokine production such as IL-12 and IFN-γ [33]. The ability of Tuftsin to be stably incorporated into liposomal membranes enables targeted delivery of antigens, making it a highly suitable adjuvant for vaccine formulations against *Candida albicans*. Tuftsin-bearing liposomes (Tuft-liposomes) have previously demonstrated notable efficacy in delivering antimicrobial agents against a variety of pathogens, including *Mycobacterium tuberculosis*, *Leishmania*, *Plasmodium*, *Candida*, and *Cryptococcus*, as well as in therapeutic models for cancers such as fibrosarcoma [32]. These properties position Tuft-liposomes as promising platforms for advanced drug and vaccine delivery systems.

In this study, we investigated a novel vaccine strategy employing Tuftsin-bearing liposomes encapsulating *C. albicans* whole cell antigens (WCAgs). By harnessing the immunostimulatory effects of Tuftsin and the delivery efficiency of liposomes, the formulation was designed to elicit both robust humoral and cellular immune responses. This approach presents a promising immunoprophylactic strategy with the potential to confer durable protection against systemic candidiasis, particularly in the context of drug-resistant fungal infections. The results of our study demonstrated that Tuft-liposomes significantly enhanced the immunogenicity of *C. albicans* antigens and effectively cleared fungal infection in immunized mice.

## 2. Materials and Methods

Avanti Polar Lipids Inc. (Alabaster, AL, USA) supplied the phosphatidylcholine and cholesterol. The C-terminal modification of Tuftsin was carried out via palmitoylation, following a previously described method [34]. Materials included Saboraud dextrose agar (SDA) and Saboraud dextrose broth (SDB) from HiMedia Co. (Mumbai, India) and antibody/IgG isotype kits from Sino Biologicals Inc. (Beijing, China). Fluconazole (FLZ) and Incomplete Freund’s Adjuvant (IFA) were obtained from Santa Cruz Biotechnology (Paso Robles, CA, USA). Kits for measuring AST, creatinine, and LDH were purchased from Abcam (Cambridge, UK).

### 2.1. Candida albicans

*Candida albicans* clinical isolate used in this study was obtained from the Microbiology Laboratory at King Fahad Hospital, Buraydah, Saudi Arabia. The isolate was originally recovered from a patient sample as part of routine diagnostic procedures and was subsequently characterized based on germtube formation and its growth on Chromogenic agar to confirm species identity.

### 2.2. In Vitro Analysis of Fluconazole’s Antifungal Potency

The antifungal potential of FLZ was assessed using an agar well diffusion assay, as previously described [35]. Plates of Sabouraud Dextrose Agar (SDA) and chromogenic agar were uniformly inoculated with *C. albicans*. Wells of 8 mm diameter were aseptically punched into the SDA plates, and each well was filled with 50 µL of fluconazole (FLZ) solution containing either 50 µg or 100 µg of the drug. Following incubation at 37 °C for 48 h, the antifungal effect was determined by the size of the inhibition zones. A well containing 50 µL of 10% DMSO served as a negative control. Additionally, FLZ activity against *C. albicans* was further evaluated using confocal microscopy. For this, the culture containing 1 × 10^5^ colony-forming units (CFUs) of *C. albicans* was incubated in SDB with FLZ at 16 µg/mL for 24 h. Cells were gently rinsed with PBS post-incubation, stained with propidium iodide, rewashed, and imaged using confocal microscopy.

### 2.3. Determining C. albicans Susceptibility Using the VITEK System

Antifungal susceptibility of the *C. albicans* strain was assessed using the VITEK 2 Compact system (bioMérieux, Craponne, France). Fresh colonies (24–48 h old) were harvested from Sabouraud Dextrose Agar and suspended in 0.9% sterile saline to a turbidity equivalent to a 0.5 McFarland standard by using a Densichek turbidity meter (bioMerieux, Craponne, France). The inoculum was loaded into the AST-YS07 VITEK antifungal susceptibility card, which includes FLZ among other antifungals. The cards were automatically sealed and incubated within the VITEK system for 18–24 h at 35 ± 1 °C, during which time microbial growth and metabolic activity were monitored. Minimum inhibitory concentration (MIC) values were interpreted according to CLSI guidelines, and the isolate was categorized as susceptible, intermediate, or resistant based on a VITEK software analysis (VITEK® 2 Systems software, version 9).

### 2.4. Determination of FLZ Efficacy Against Biofilm Formation

To evaluate the effects of FLZ on preformed *C. albicans* biofilms, a standard biofilm quantification assay, a suspension of *C. albicans* (1 × 10^6^ CFU/mL) was dispensed (100 µL/well) into a 96-well microtiter plate followed by incubation for 24 h to allow biofilm formation. Consequently, the used SDB was carefully replaced with fresh SDB containing FLZ at concentrations of 8, 16, and 32 µg/mL. After 24 h of treatment, non-adherent cells were eliminated by washing with PBS. Following a 20 min stain with 0.1% crystal violet, biofilms were washed thoroughly with PBS and left to air-dry. Biofilm-associated dye was solubilized with 100 µL of 95% ethanol, and absorbance was measured at 595 nm.

### 2.5. Mice

BALB/c female mice were supplied by the animal house of the College of Applied Medical Sciences, Qassim University. Ethical clearance was secured from Research Ethics at the Deanship of Graduate Studies and Scientific Research, Qassim University (Ethical Approval # 24-94-03, dated 9 June 2024). All efforts were made to minimize animal sufferings and reduce the number of animals used. The animals were housed under standard laboratory conditions with appropriate care, feeding, and monitoring throughout the experimental period.

### 2.6. FLZ Intervention in Mice Infected with C. albicans

In the animal model, FLZ was administered intraperitoneally on days 1, 3, and 5 post-infection to mimic an intermittent therapeutic regimen, consistent with protocols established in murine *C. albicans* infection studies [35]. This schedule allowed for an evaluation of both early and cumulative drug effects while adhering to ethical and humane animal handling standards. Four groups of mice (*n* = 10 each) were established: (1) normal saline, (2) FLZ 5 mg/kg, (3) FLZ 10 mg/kg, and (4) FLZ 20 mg/kg. To evaluate FLZ efficacy, survival and kidney fungal burden were monitored using established procedures [35]. Following euthanasia on day 7 post-infection, the kidneys were collected under sterile conditions, homogenized, and serially diluted. Infection severity was assessed by plating homogenates on SDA and counting the resulting CFUs. This experimental design enabled a comparative analysis of FLZ’s therapeutic efficacy in mitigating systemic *C. albicans* infection.

### 2.7. Whole Cell Antigens (WCAgs) of C. albicans

After culturing *C. albicans* on SDA for 48 h, the cells were harvested and washed three times with ice-cold 50 mM PBS (pH 7.4) to remove residual media components. This washing step ensures that the final antigen preparation is free from culture medium proteins that could interfere with immune responses. The washed cell pellet was then resuspended in lysis buffer consisting of 50 mM PBS supplemented with 1% Triton X-100 and a commercially available protease inhibitor cocktail (Roche, Indianapolis, IN, USA). The inclusion of Triton X-100 facilitated membrane disruption, whereas the protease inhibitors prevented the degradation of antigenic proteins during lysis. Lysis was performed by probe sonication on ice (3–5 cycles of 30 s with 30 s intervals) to ensure thorough disruption of yeast cells while minimizing protein denaturation. Following lysis, the suspension was centrifuged at 10,000× *g* for 15 min at 4 °C. This step separated the soluble antigenic fraction (supernatant) from cell debris. The supernatant containing the WCAgs was collected, quantified using a bicinchoninic acid (BCA) protein assay, and stored at −80 °C until use in the vaccine formulation.

### 2.8. Preparation and Characterization of Dried-Reconstituted Vesicles (DRVs)

DRVs were selected due to their proven utility in stabilizing liposomes for storage and enabling efficient rehydration with aqueous antigens [36]. A lipid mixture composed of egg phosphatidylcholine (49 µmol) and cholesterol (21 µmol) was dissolved in a 2:1 chloroform–methanol solvent system. Separately, palmitoylated Tuftsin was dissolved in methanol and added to the lipid phase. The solvent was removed using rotary evaporation under reduced pressure at 37 °C to create a thin lipid film. The lipid film was then hydrated with an aqueous solution of WCAgs (1 mg/mL in PBS) under constant vortexing to form multilamellar vesicles. This suspension was subjected to three freeze–thaw cycles (−80 °C for 5 min; 37 °C for 5 min) to enhance encapsulation efficiency. The resulting vesicle dispersion was lyophilized to obtain the dried form of DRVs. For reconstitution, the dry powder was resuspended in sterile 0.9% NaCl (normal saline) at room temperature, followed by gentle vortexing to facilitate uniform vesicle formation. Following rehydration, the liposome suspension was centrifuged at 25,000× *g* for 30 min at 4 °C to pellet the liposomes and separate unencapsulated antigens, which remain in the supernatant. The pellet containing WCAg-loaded Tuftsin-liposomes was carefully resuspended in sterile PBS for use in subsequent experiments. This method ensures recovery of intact vesicles and excludes free WCAgs from the final vaccine formulation. Liposomal particle size and polydispersity index (PDI) were measured using the dynamic light scattering (DLS) on a Malvern Nano Zeta Sizer (Malvern Instruments Ltd., Malvern, Worcestershire, UK). The stability of liposomal vaccine was assessed by storing the formulation at 4 °C for a week.

### 2.9. Estimation of WCAgs Encapsulated in Tuft-Free and Tuft-Bearing Liposomes

The loading of WCAgs into Tuft-free and Tuftsin-bearing liposomes was quantified using a protein estimation kit. Unencapsulated WCAgs was removed by centrifugation, and the protein concentration was measured to calculate the amount of WCAgs retained within the liposomes.

### 2.10. In Vitro Release of WCAgs from Tuftsin-Free and Tuftsin-Bearing Liposomes

To evaluate the stability of the liposomal formulations, an in vitro release study was conducted at 4 °C over 7 days. Equal volumes of the Tuft-lip-WCAg and Lip-WCAg formulations containing known concentrations of WCAgs were placed into pre-wetted dialysis bags. Each bag was immersed in 5 mL of phosphate-buffered saline (PBS, pH 7.4) at 4 °C. At designated time points (0, 1, 2, 3, 5, and 7 days), 500 µL of the external PBS medium was withdrawn for analysis and replaced with an equal volume of fresh PBS. The amount of WCAgs released at each time point was quantified using a BCA protein assay. Cumulative antigen release was calculated and expressed as a percentage of the total encapsulated protein.

### 2.11. Immunization

**Figure 1:**
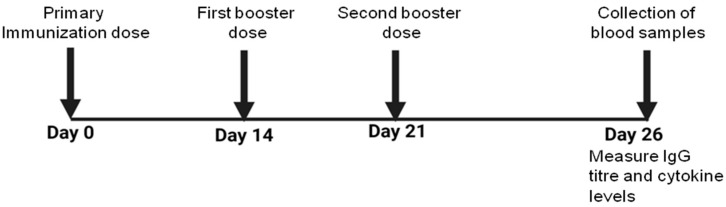
The schedule of immunization with various vaccine formulations in the mice safety assessment of vaccine formulations.

Mice in each group were immunized subcutaneously with 25 µg of WCAg formulated in either Tuftsin-bearing liposomes or Tuftsin-free liposomes. A separate control group received WCAgs emulsified with Incomplete Freund’s Adjuvant (IFA) in equivalent amounts. Two booster doses of the respective vaccine formulations were administered on days 14 and 21 post-primary immunization (Figure 1). The experimental design included the following groups: (1) saline, (2) sham-liposomes, (3) sham-Tuft-liposomes, (4) IFA-WCAgs, (5) Lip-WCAgs, and (6) Tuft-lip-WCAgs.

Blood samples were obtained from three randomly selected mice per group on day 5 following the final booster. Measurements of serum aspartate transaminase, creatinine, and lactate dehydrogenase levels were performed by using specific kits to assess potential hepatic, renal, and cardiac toxicity, respectively.

### 2.12. Status of WCAg-Specific Antibody Level and IgG Isotypes

Serum samples were collected from vaccinated mice on day 26 to measure total antibody titer, IgG1, and IgG2a isotype levels. ELISA plates were coated overnight at 4 °C with 100 µL/well of WCAgs at a concentration of 5 µg/mL in carbonate-bicarbonate buffer (pH 9.6). Plates were blocked with 5% defatted milk in PBS overnight at 4 °C. Serial dilutions of mouse sera were added and incubated for 1 h at 37 °C. After washing, horseradish peroxidase (HRP)-conjugated goat anti-mouse IgG, IgG1, or IgG2a secondary antibodies (diluted 1:10,000) were added and incubated for 1 h at 37 °C. TMB substrate was added, and the reaction was stopped with 2N sulfuric acid. Absorbance was read at 450 nm using a microplate reader.

### 2.13. BrdU-Based T Cell Proliferation Assay

To assess antigen-specific T cell responses, the splenocytes (lymphocytes derived from the spleens of immunized mice) were isolated on day 26 post-immunization and used in a BrdU-based proliferation assay using the Cell Titer 96 ELISA kit (Abcam, Cambridge, UK). Splenocytes were cultured in 96-well flat-bottom plates at 1 × 10^5^ cells/well in RPMI-1640 medium supplemented with 10% FBS, 1% penicillin/streptomycin, and 2 mM L-glutamine. The Cells were stimulated in triplicate with WCAgs at 8 µg/mL for 48 h [28]. Concanavalin A (Con A, 2 µg/mL) served as a positive control, whereas ovalbumin (8 µg/mL) was used as a non-relevant antigen control. After incubation, 10 µM BrdU was added for 16 h. The cells were fixed, and BrdU incorporation was measured using a BrdU ELISA kit (Abcam) per manufacturer’s protocol. Absorbance was read at 450/550 nm. The stimulation index (SI) was calculated by dividing the absorbance of antigen-stimulated wells by that of unstimulated controls.

### 2.14. Analysis of IFN-γ and IL-4 Cytokine Profiles

Splenocytes were isolated from immunized mice on day 26, five days after the final booster. Spleens were aseptically harvested and passed through a 70 µm cell strainer into RPMI-1640 medium containing 10% FBS, 1% penicillin-streptomycin, and 2 mM L-glutamine. Red blood cells were lysed using an ACK lysis buffer, and cells were washed, centrifuged at 1500× *g* for 10 min, and resuspended in fresh medium. Viable splenocytes were counted by trypan blue exclusion. The cells were seeded at 1 × 10^6^ cells/well in 24-well plates and stimulated with 1 µg/mL of WCAgs. Control wells received either medium alone or ovalbumin (1 µg/mL). Cultures were incubated at 37 °C in 5% CO_2_ for 48 h. Supernatants were then collected, centrifuged at 1500× *g* for 10 min at 4 °C to remove debris, and stored at −20 °C. The levels of IFN-γ and IL-4 were measured using commercial mouse-specific ELISA kits following the guidelines of the manufacturer (Abcam, Cambridge, UK).

### 2.15. Infection of Vaccinated Mice with C. albicans

*C. albicans* was initially cultured in SDB at 37 °C for 24 h to promote optimal fungal growth. The culture was then centrifuged at 3000× *g* for 10 min at room temperature to pellet the fungal cells. The supernatant was discarded, and the cell pellet was washed twice with sterile PBS. To ensure a uniform suspension, the final pellet was gently resuspended in sterile PBS by vortexing. Fungal cell concentration was determined using a hemocytometer, and the inoculum was adjusted to a final concentration of 7 × 10^5^ viable cells per 200 µL, ensuring consistent delivery across all mice. On day 28, one week following the final booster dose, each mouse was injected intravenously with 200 µL of the prepared *C. albicans* suspension, corresponding to 7 × 10^5^ viable yeast cells per animal.

### 2.16. To Assess the Prophylactic Effectiveness of Tutf-Lip-WCAg or Lip-WCAg Vaccine Formulations Against the Systemic Candidiasis

The protective efficacy of immunization against *C. albicans* was determined by tracking the survival and measuring fungal burden in the homogenized kidney tissues of infected mice. The intensity of candidiasis was evaluated after 7 days of infection, and the kidneys were extracted and homogenized. Tissue homogenate dilutions were plated onto SDA to measure *C. albicans* colony counts. Furthermore, to confirm complete pathogen clearance, the kidney tissues were collected on day 40 post-infection from mice that survived the entire observation period. Fungal load was assessed in these samples to evaluate the protection conferred by the vaccine.

### 2.17. Statistical Analyses

Survival data were analyzed by constructing Kaplan–Meier curves and applying the log-rank (Mantel–Cox) test using GraphPad Prism (v5.0). Differences in immunological parameters and fungal burden among groups were evaluated via one-way ANOVA followed by Tukey’s multiple comparison test. A *p*-value < 0.05 indicated statistical significance.

## 3. Results

### 3.1. FLZ Exhibited No Effective Activity Against C. albicans

**Figure 2:**
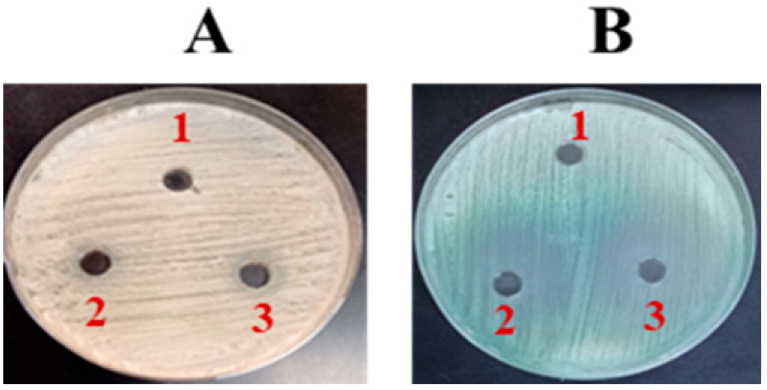
The activity of FLZ on (**A**) SDA and (**B**) Chromogenic agar. (1) Control, (2) FLZ-50 µg/mL, (3) FLZ-100 µg/mL.

The effectiveness of FLZ against *C. albicans* was measured using an agar well diffusion assay. The results indicated that FLZ exhibited no significant impact on *C. albicans* growth, as evidenced by the absence of a clear zone of inhibition on SDA (Figure 2A). This suggests that *C. albicans* may exhibit resistance to FLZ under the tested conditions. However, on chromogenic agar, a slight fungistatic effect was observed (Figure 2B).

**Figure 3:**
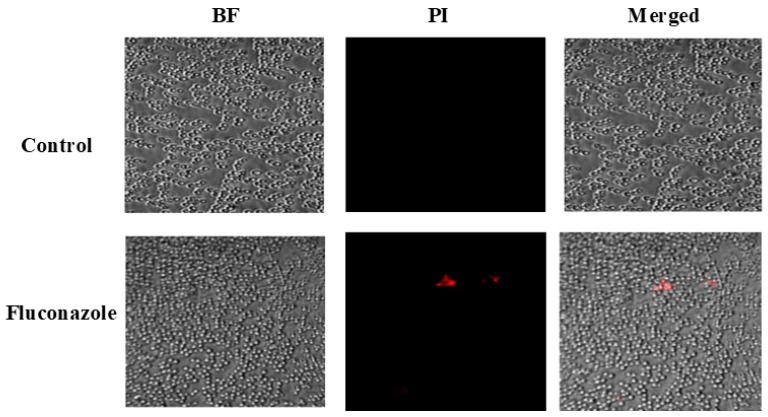
Confocal microscopy analysis of *C. albicans* viability following treatment with FLZ using propidium iodide (PI) staining.

To evaluate the impact of FLZ on fungal cell viability, confocal microscopy combined with PI staining was performed. Figure 3 shows that FLZ treatment caused only a minor elevation in PI-positive cells (shown in red color), indicating that the drug did not effectively compromise *C. albicans* membrane integrity.

According to the VITEK susceptibility testing results, the current strain of *C. albicans* exhibited an MIC of 64 µg/mL for FLZ. This MIC value suggests that the strain displays potential resistance to FLZ, depending on clinical breakpoints.

### 3.2. FLZ Did Not Eradicate the Preformed Biofilm of C. albicans

**Figure 4:**
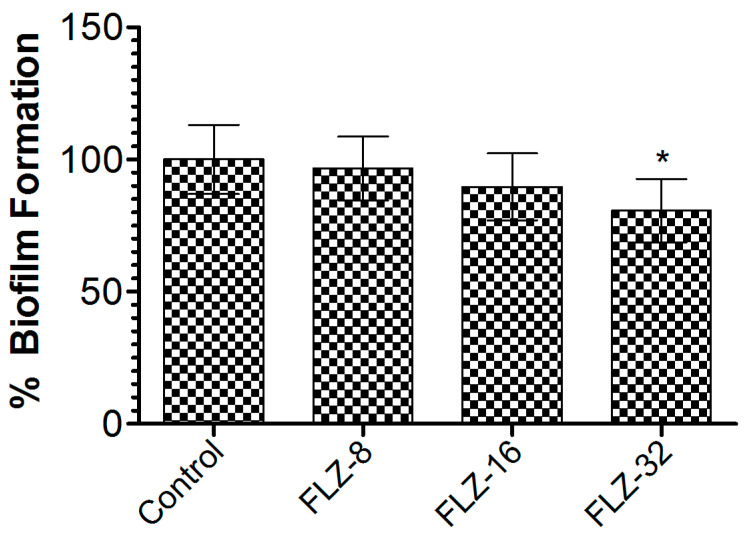
Effect of FLZ (8, 16, and 32 µg/mL) on *C. albicans*. Data are presented as mean ± SD based on three independent measurements. A *p* value < 0.05 was considered significant. * (*p* < 0.05) infected control vs. FLZ-32 µg/mL.

To assess FLZ’s effectiveness against *C. albicans* biofilms, the preformed biofilms were treated with increasing concentrations (8, 16, and 32 µg/mL). At 32 µg/mL, FLZ achieved a 21.7% reduction in biofilm biomass compared to the vehicle-treated group (Figure 4), with statistical significance (*p* < 0.05). The limited biofilm disruption suggests that the *C. albicans* biofilm exhibited resistance mechanisms that diminish FLZ’s efficacy, highlighting the need for alternative strategies to enhance antifungal activity.

### 3.3. FLZ Does Not Effectively Treat C. albicans Infection in Mice

**Figure 5:**
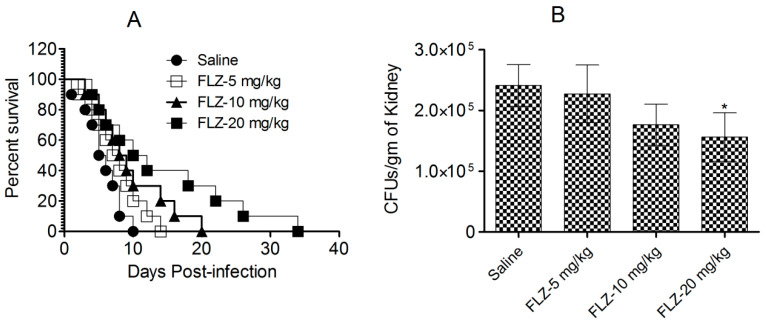
FLZ did not demonstrate efficacy against *C. albicans* in mice. (**A**) Survival analysis of infected mice following treatment with various FLZ doses. (**B**) On day 7, kidneys from three mice were homogenized, and the homogenates were cultured on SDA to assess fungal growth. Fungal load was calculated and reported as the mean ± S.D. from three values. (* *p* < 0.05) Saline vs. FLZ-20 mg/kg.

Mice challenged with *C. albicans* were administered FLZ, and their survival was assessed for 40 days after infection (Figure 5A). The highest dose of FLZ (20 mg/kg) extended the median survival time to 16 days, compared to only 6 days in the control group (*p* < 0.001). However, despite this initial improvement, all FLZ-treated mice succumbed to the infection within 40 days (Figure 5A), indicating that FLZ provided only a temporary survival benefit without achieving long-term cure.

In addition to a survival analysis, the antifungal efficacy of FLZ was evaluated by quantifying fungal burden in kidney tissue on day 7 post-infection (Figure 5B). The untreated control mice exhibited the highest fungal load, with an average of 241,296 ± 34,410 CFUs per gram of kidney tissue. By comparison, mice administered with the highest FLZ dose (20 mg/kg) showed lower fungal load, containing 156,283 ± 40,005 CFUs, which was statistically insignificant (*p* > 0.05). Likewise, mice receiving 10 mg/kg and 5 mg/kg FLZ similarly retained fungal burdens of 227,485 ± 27,321 and 176,584 ± 33,815 CFUs, respectively (Figure 5B). These findings suggest that while FLZ slightly reduced fungal colonization in kidney tissue, its effect was insufficient in clearing the infection from the infected mice.

### 3.4. Characterization and Entrapment Efficiency of WCAgs in Tuft-Bearing and Tuftsin-Free Liposomes

The polydispersity index (PDI) values for Tuft-lip-WCAgs and Lip-WCAgs were found to be 0.342 and 0.365, respectively. These values indicate a moderately uniform size distribution, which is considered acceptable for nanoparticulate drug delivery systems. A PDI value (0 to 1) reflects the colloidal stability of formulation, with values below 0.5 generally indicative of a homogenous population. A DLS analysis revealed that the particle sizes of both formulations ranged from 60 to 220 nm, which falls within the optimal size range. The entrapment efficiency of *C. albicans* whole cell antigens (WCAgs) was 47.2% for Tuft-lip-WCAgs and 45.8% for Lip-WCAgs, suggesting that Tuftsin conjugation did not negatively affect antigen loading capacity.

The stability of the Tuft-lip-WCAg formulation was assessed at 4 °C for a week. Under these conditions, the structural integrity of the liposomes was detected to be preserved, showing no aggregation. Palmitoylated Tuftsin was incorporated into the bilayer, which protects the peptide from degradation during refrigerated storage. To monitor physical stability, we assessed the PDI value immediately after reconstitution and following storage at 4 °C over the period of a week. No significant changes in size or PDI were observed during the storage period, supporting the colloidal stability of the formulation. Additionally, the dried-reconstituted vesicle (DRV) approach used in this study supports improved shelf stability upon rehydration.

### 3.5. Tuftsin-Bearing Liposomes Exhibited Reduced Release of WCAgs at 4 °C

**Figure 6:**
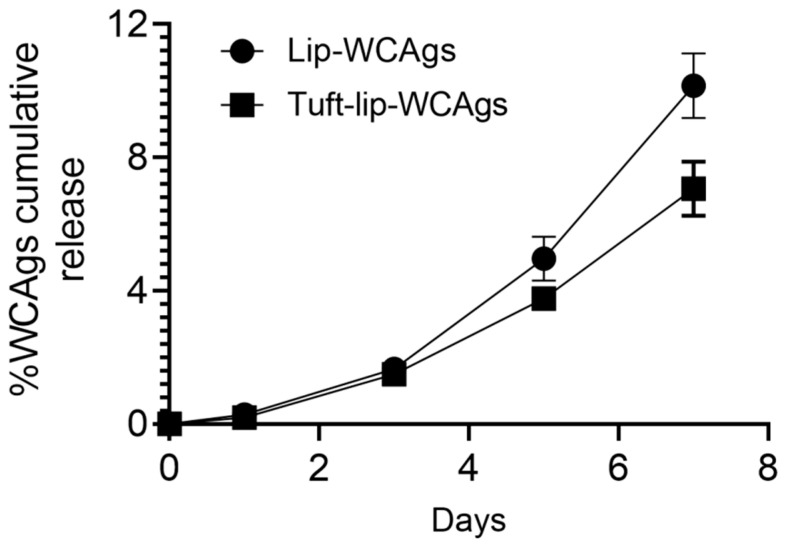
The cumulative release of WCAgs from Tuft-lip-WCAg and Lip-WCAg formulations over 7 days at 4 °C. At designated time points, aliquots from the release medium were collected and analyzed using a BCA protein assay. Tuftsin-bearing liposomes showed a slower release rate of WCAgs, compared to Tuftsin-free liposomes, indicating enhanced antigen retention. Data represent the mean ± S.D. of three independent measurements.

To evaluate the antigen retention capacity of the liposomal formulations, the cumulative release of WCAgs was assessed over a 7-day period at 4 °C. As illustrated in Figure 6, the Tuft-lip-WCAg formulation demonstrated minimal lower release of antigens compared to the Lip-WCAg formulation. By day 7, the Tuftsin-free liposomes had released approximately 10% of the encapsulated WCAgs, while the Tuftsin-bearing liposomes released only about 7%. This reduced antigen leakage suggests enhanced membrane stability in the presence of Tuftsin, supporting the short-term physical integrity of the Tuft-lip-WCAg formulation under refrigerated storage conditions.

### 3.6. Tuft-Lip-WCAg or Lip-WCAg Vaccine Formulations Exhibited No Detectable Toxicity

**Figure 7:**
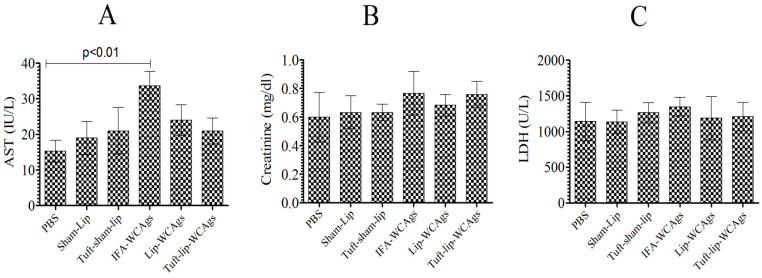
Tuft-lip-WCAg immunization was well tolerated and free of toxic effects. The amounts of (**A**) AST: PBS vs. IFA-WCAgs (*p* < 0.01), (**B**) creatinine, and (**C**) LDH were investigated in the serum of immunized mice. The data were was calculated as the mean ± S.D. from three measurements.

Mice, vaccinated with various formulations, behaved normally and showed no vaccine-associated toxic signs (Figure 7A–C). Mice treated with Tuft-lip-WCAgs and Lip-WCAgs showed AST levels (24 ± 4.4 and 21 ± 3.3 IU/L, respectively) that were consistent with those of the PBS control group (15.5 ± 3.3 IU/L), indicating no evidence of liver toxicity (Figure 7A). The creatinine levels measured in Tuft-lip-WCAg- and Lip-WCAg-immunized mice were 0.68 ± 0.76 mg/dL and 0.76 ± 0.91 mg/dL, respectively. These values fall within the normal reference range, suggesting preserved kidney function following immunization (Figure 7B). To evaluate potential cardiac toxicity, LDH levels were measured in immunized mice. The LDH concentrations observed in the Lip-WCAg and Tuft-lip-WCAg groups (1199 ± 120 U/L and 1211 ± 780 U/L, respectively) were not significantly different from those in the sham-liposome group (1143 ± 108 U/L), supporting the cardiac safety of both formulations (Figure 7C).

### 3.7. Superior Antigen-Specific Immune Responses Were Induced by Tuft-Lip-WCAgs

**Figure 8:**
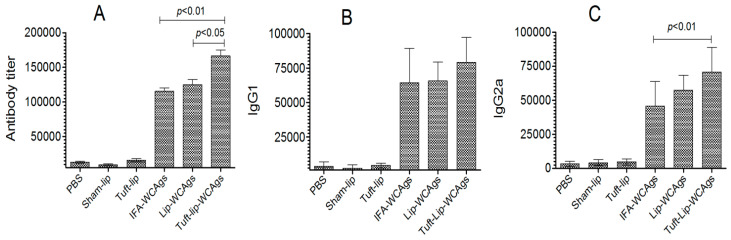
Tuft-lip-WCAgs elicited a significant increase in WCAg-specific antibody production, surpassing the responses induced by other formulations. (**A**) Total antibody: IFA-WCAgs vs. Tuft-lip-WCAgs (*p* < 0.01); Lip-WCAgs vs. Tuft-lip-WCAgs (*p* < 0.05), (**B**) IgG1 and (**C**) IgG2a: IFA-WCAgs vs. Tuft-lip-WCAgs (*p* < 0.01). The results are shown as mean ± S.D. from three separate experiments, and the data were analyzed by one-way ANOVA with Tukey’s post hoc test.

Immunization with Tuft-lip-WCAgs elicited the highest antibody production relative to Lip-WCAgs and IFA-WCAgs (*p* < 0.05 and *p* < 0.01, respectively). The antibody titer was found to be 166,667 ± 14,434 in Tuft-lip-WCAgs, whereas Lip-WCAg-vaccinated mice had 125,000 ± 12,500 (Figure 8A). Moreover, the titers of IgG isotypes were examined in mice vaccinated with Tuft-lip-WCAgs or Lip-WCAgs or IFA-WCAgs. No significant difference was noted in IgG1 isotype among Tuft-lip-WCAg-, Lip-WCAg-, and IFA-WCAg-immunized mice, whereas the IgG2a was detected to be the highest in Tuft-lip-WCAg-immunized mice (Figure 8B,C).

### 3.8. Tuft-Lip-WCAgs Induced Higher Proliferation of T Lymphocytes

**Figure 9:**
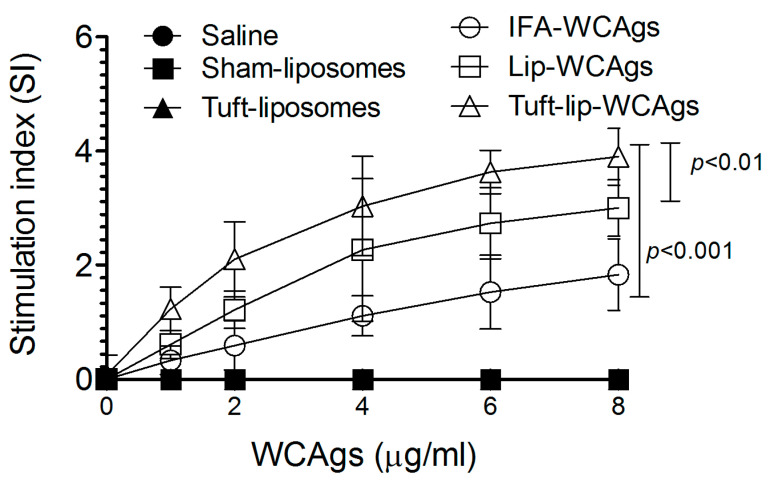
Tuft-lip-WCAgs significantly enhanced the proliferation of T lymphocytes compared to other vaccine formulations. Data represent the mean ± S.D. of T cell stimulation indices from three mice per group. The control groups (PBS, sham-lip, and Tuft-lip) showed negligible responses to the WCAg stimulation and are included for reference.

Upon antigenic stimulation at 8 µg/mL, T cells from Tuft-lip-WCAg-immunized mice demonstrated significantly greater proliferation than those from mice vaccinated with Lip-WCAgs (*p* < 0.01) or IFA-WCAgs (*p* < 0.001). T lymphocytes from Tuft-lip-WCAg-immunized mice showed a stimulation index of 3.9 ± 0.84, followed by Lip-WCAgs (3.0 ± 0.61), with the lowest in IFA-WCAg-immunized mice (1.83 ± 0.41), indicating enhanced T cell activation with the Tuftsin-incorporated formulation (Figure 9).

### 3.9. Immunization with Tuft-Lip-WCAgs Elicited Higher IFN-γ and IL-4 Secretion

**Figure 10:**
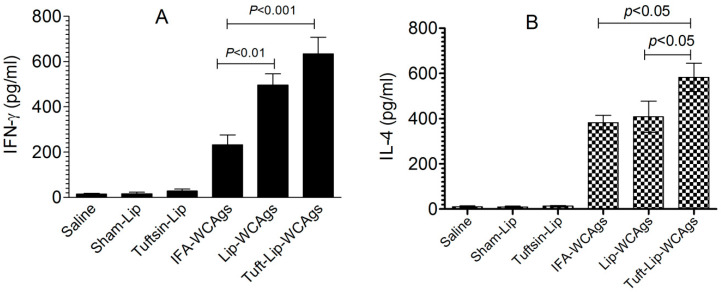
Tuft-lip-WCAg vaccination resulted in elevated levels of both IFN-γ and IL-4. (**A**) IFN-γ levels: significant differences were observed between IFA-WCAgs and Lip-WCAgs (*p* < 0.01), and between IFA-WCAgs and Tuft-lip-WCAgs (*p* < 0.001). (**B**) IL-4 levels: Tuft-lip-WCAgs differed significantly from both IFA-WCAgs and Lip-WCAgs (*p* < 0.05 for each comparison). Cytokine levels in the culture supernatant were quantified and expressed as mean ± S.D.

Tuft-lip-WCAg immunization induced a stronger IFN-γ response in spleen cells (634 ± 128 pg/mL) than Lip-WCAg immunization (496 ± 86 pg/mL), reflecting a more potent Th1-mediated immune response (Figure 10A). In contrast, the spleen cells from IFA-WCAg-immunized mice produced 232 ± 76 pg/mL of IFN-γ. IL-4 was detected to be 582 ± 82 pg/mL in the Tuft-lip-WCAg-vaccinated mice, which is higher than the 408 ± 69 pg/mL detected in mice receiving Lip-WCAgs (Figure 10B).

### 3.10. Immunization with Tuft-Lip-WCAg Imparted Highest Resistance to Mice Against C. albicans

**Figure 11:**
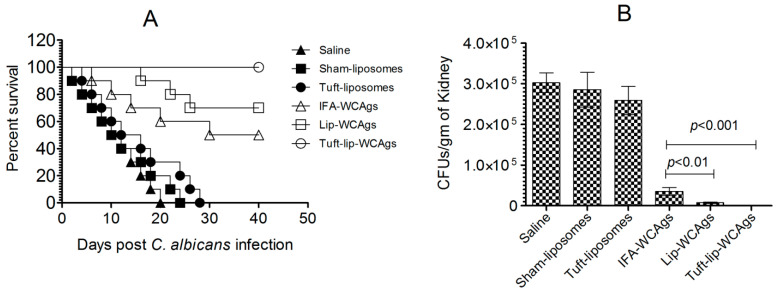
Tuft-lip-WCAg vaccination conferred the strongest resistance to *C. albicans* infection in mice. (**A**) Tuft-lip-WCAg-, Lip-WCAg-, and IFA-WCAg-immunized mice were challenged with *C. albicans* following the protocol described in the methods. (Tuft-Lip-CAgs vs. IFA-CAgs, *p* = 0.0115). (**B**) On the seventh day after infection, kidneys from three mice were harvested and analyzed for fungal burden. IFA-WCAgs and Lip-WCAgs (*p* < 0.01), IFA-WCAgs and Tuft-lip-WCAgs (*p* < 0.001). All data are expressed as the mean ± S.D. derived from three independent experimental values.

Tuft-lip-WCAg vaccination resulted in superior survival after *C. albicans* infection compared to the Lip-WCAg or IFA-WCAg groups. A survival analysis showed that all mice immunized with Tuft-lip-WCAgs survived the challenge, compared to 70% survival in the Lip-WCAg group and 50% in the IFA-WCAg group (Figure 11A). Tuft-Lip-WCAg vaccination showed superior protection against *C. albicans* infection than the immunization with Lip-CAgs or IFA-WCAgs.

Among the groups tested, Tuft-lip-WCAg vaccination resulted in the greatest reduction in *C. albicans* proliferation, surpassing the efficacy observed with Lip-WCAgs or IFA-WCAgs (Figure 11B). The kidney tissue from unimmunized mice in the control groups infected with *C. albicans* contained highly elevated fungal burden. On the other hand, there was remarkably reduced fungal load in mice that received Tuft-Lip-WCAgs or Lip-WCAgs or IFA-WCAgs as compared to their corresponding control groups (*p* < 0.001). Tuft-lip-WCAg-vaccinated mice contained 556 ± 197 CFUs/gram of *C. albicans* in their kidney tissue, which is significantly lower than the 35,108 ± 10,141 CFUs/gram in the mice vaccinated with IFA-WCAgs (*p* < 0.001). In the end, the surviving mice were screened for fungal load on day 40 and were found to be free of *C. albicans* infection as there was no growth on SDA plates cultured with the kidney tissue homogenates of the mice.

## 4. Discussion

Liposomes serve as reliable vaccine carriers, efficiently directing antigens to APCs like dendritic cells and macrophages to enhance immune activation [37,38]. They facilitate antigen presentation, promoting CD8+ and CD4+ T cell activation. In the current investigation, we explored the immunoadjuvant potential of Tuftsin-modified liposomes, leveraging Tuftsin’s ability to activate phagocytic cells via its interaction with surface receptors on macrophages and dendritic cells. Before evaluating the vaccine formulations, the antifungal efficacy of FLZ was assessed as a comparative reference. In in vitro assays, FLZ exhibited minimal inhibitory activity against *C. albicans* on SDA plates and only a slight fungistatic effect on chromogenic agar. Confocal microscopy with PI staining confirmed that FLZ failed to induce significant fungal cell death, supporting the notion of azole resistance in the tested strain. The VITEK system analysis confirmed this resistance, reporting an MIC of 64 µg/mL close to the resistance threshold defined by CLSI [39].

Fluconazole also showed limited efficacy in vivo as all treated mice eventually succumbed by day 40. The fungal burden analysis further revealed only modest reductions in kidney CFUs as compared to untreated controls, and the differences were statistically insignificant. These results indicate that while FLZ can delay disease progression, it fails to achieve fungal clearance, especially in drug-resistant strains. Additionally, FLZ demonstrated poor performance against biofilm-associated *C. albicans*. At its highest concentration (32 µg/mL), FLZ only reduced biofilm biomass by ~21%, reinforcing earlier observations that *C. albicans* biofilms confer a protective phenotype that limits drug penetration and efficacy [27]. These findings underscore the urgent need for alternative strategies, particularly immunoprophylactic approaches like vaccines.

The stability of the Tuft-lip-WCAg formulation is a critical factor for its translational potential as a vaccine candidate. In this study, the formulation was stored at 4 °C to reflect standard refrigerated storage conditions. An antigen release analysis over a 7-day period revealed that Tuftsin-bearing liposomes exhibited lower antigen leakage than Tuftsin-free liposomes. In addition, the polydispersity index (PDI) of Tuft-lip-WCAgs showed moderately uniform particle population, supporting the formulation’s colloidal stability. The combination of low antigen release and acceptable PDI under refrigerated conditions confirms the short-term physical and chemical stability of the Tuft-lip-WCAg formulation. These results support its suitability for further development, while also highlighting the need for future studies addressing long-term storage stability and stress testing under varying environmental conditions.

We, therefore, developed a Tuftsin-bearing liposomal formulation encapsulating WCAgs, termed Tuft-lip-WCAgs, and assessed its immunogenicity and protective efficacy in a mouse model. Our findings revealed that Tuft-lip-WCAgs induced a significantly stronger immune response than Lip-WCAgs or IFA-WCAgs. Notably, toxicity profiling showed that neither Tuft-lip-WCAgs nor Lip-WCAgs induced any signs of hepatotoxicity, nephrotoxicity, or cardiotoxicity, as indicated by normal AST, creatinine, and LDH values in the vaccinated mice, aligning with previous safety assessments of liposomal vaccines [29]. However, these parameters alone may not fully capture subclinical or organ-specific toxicities. Future studies should incorporate more comprehensive histopathological evaluations and longer-term toxicity monitoring to validate the formulation’s safety profile.

Tuft-lip-WCAg immunization elicited significantly elevated antibody levels, with total antibody titers reaching 166,667 ± 14,434 as compared to 125,000 ± 12,500 in the Lip-WCAg group. More importantly, the IgG2a isotype was predominant in Tuft-lip-WCAg-immunized mice, consistent with a Th1-biased immune response, which is known to mediate the activation of complement and Fc receptor-mediated pathways [40]. While the IgG1 levels remained comparable across groups, the higher IgG2a levels support the adjuvant role of Tuftsin in skewing the immune response in favor of protective Th1-type immunity. The cell-mediated responses were also markedly enhanced. T lymphocytes from Tuft-lip-WCAg-immunized mice exhibited significantly enhanced proliferation in mice (Figure 9). These findings align with previous studies demonstrating Tuftsin’s ability to stimulate T cell responses and rejuvenate immune function in immunocompromised models [41,42].

Cytokine profiling revealed an effective Th1/Th2 mixed response in the Tuft-lip-WCAg group, as evidenced by elevated IFN-γ and IL-4 levels as compared to the lower levels in the other immunized groups. IFN-γ had a pivotal role in antifungal defense by activating phagocytes and enhancing opsonization and antigen presentation [43], while IL-4 plays a crucial role in promoting B cell differentiation and boosting antibody responses [44]. Together, these responses suggest well-balanced and comprehensive immune activation, essential for effective protection against pathogens like *C. albicans*.

Importantly, the protective efficacy of Tuft-lip-WCAgs was confirmed through survival and fungal burden analyses. Tuft-lip-WCAg-vaccinated mice achieved a 100% survival rate following a lethal *C. albicans* challenge, compared to the 70% and 50% survival rates in the Lip-WCAg and IFA-WCAg groups, respectively. Fungal load in kidney tissues was considerably reduced in the Tuft-lip-WCAg group (556 ± 197 CFUs/g), significantly lower than in the IFA-WCAg group (35,108 ± 10,141 CFUs/g), indicating superior fungal clearance. Furthermore, no fungal growth was detected in the kidney tissues on day 40, highlighting the short-term protection conferred by the Tuft-lip-WCAg formulation and supporting the need for further studies to assess the durability of immune protection over longer periods.

These results support the immunopotentiating potential of Tuftsin-bearing liposomes and highlight their promise as a platform for further development of antifungal vaccine candidates. Previous studies have shown that Tuftsin enhances the immunogenicity of viral structural proteins [41,42], and our findings extend this utility to vaccine-induced protection against drug-resistant systemic candidiasis. Unlike other vaccine candidates such as NDV-3A, which is based on the Als3p adhesin and has shown efficacy in preventing recurrent vulvovaginal candidiasis (VVC) in clinical trials, or Sap2-based vaccines that target secreted aspartyl proteinases [21,23] , the Tuft-lip-WCAg formulation employs whole cell antigens encapsulated in Tuftsin-bearing liposomes to elicit a broader and more balanced immune response. Whereas NDV-3A and similar subunit vaccines focus on specific virulence factors, the Tuft-lip-WCAg formulation targets a wider range of *Candida* antigens and leverages the immunostimulatory effect of Tuftsin to enhance antigen-specific immune responses, resulting in full protection against systemic candidiasis, as evidenced by the 100% survival and complete clearance of the pathogen from kidney tissue, a representative target organ for fungal dissemination.

## 5. Conclusions

In conclusion, the incorporation of Tuftsin increases the immunoadjuvant potential of liposomes. It is evident from the results of this study that the antibody and IgG2a isotype levels in the serum of mice vaccinated with Tuft-lip-WCAgs increased compared with those in mice vaccinated with Lip-WCAgs or IFA-WCAgs. Tuft-lip-WCAg immunization also resulted in greater T cell proliferation. Moreover, Tuft-lip-WCAg vaccination imparted the strongest resistance to *C. albicans*, as revealed by the high survival rate coupled with the lower fungal burden. On the whole, the outcomes of this research support further investigation and development of the Tuft-lip-WCAg vaccine formulation as a promising strategy to control *C. albicans* infection, particularly in light of the complex host-pathogen interactions and the known challenges of translating antifungal vaccines to clinical use in humans.

## Data Availability

All the relevant data have been provided in the paper.

## Abbreviations

MIC	Minimum Inhibitory Concentration
WCAgs	Whole Cell Antigens
Tuft-lip-WCAgs	Tuftsin-Bearing Liposomes Loaded with Whole Cell Antigens
FLZ	Fluconazole
PBS	Phosphate-Buffered Saline
ELISA	Enzyme-Linked Immunosorbent Assay
IFN-γ	Interferon-gamma
IL-4	Interleukin-4
AST	Aspartate Transaminase
LDH	Lactate Dehydrogenase
DLS	Dynamic Light Scattering
